# hsa-miR-33-5p as a Therapeutic Target Promotes Apoptosis of Breast Cancer Cells *via* Selenoprotein T

**DOI:** 10.3389/fmed.2021.651473

**Published:** 2021-04-27

**Authors:** Wei Zhuang, Jianhui Liu, Wenjin Li

**Affiliations:** ^1^Department of Laboratory, Jinan People's Hospital Affiliated to Shandong First Medical University, Jinan, China; ^2^Department of Radiotherapy, Yantai Yuhuangding Hospital, Yantai, China; ^3^Department of Breast, Linyi Cancer Hospital, Linyi, China

**Keywords:** breast cancer, hsa-miR-33-5p, Selenoprotein T, apoptosis, therapeutic target

## Abstract

**Objective:** Increasing evidence suggests that microRNA (miRNA) participates in regulating tumor cell apoptosis. We aimed to observe the effect of hsa-miR-33-5p on the apoptosis of breast cancer cells and to explore its regulatory relationship with selenoprotein T (SelT).

**Methods:** RT-qPCR was used to examine the expression of hsa-miR-33-5p and SelT both in breast cancer tissues and cells. MCF-7 and MDA-MB-231 cells were transfected with hsa-miR-33-5p mimics or si-SelT. Then, a flow cytometry assay was carried out to examine the apoptosis of cells. Furthermore, SelT and apoptosis-related proteins including caspase-3, caspase-8, caspase-9, Bax, and Bcl-2 were detected *via* RT-qPCR and western blot. A luciferase reporter assay was utilized for assessing whether SelT was targeted by hsa-miR-33-5p.

**Results:** Downregulated hsa-miR-33-5p was found both in breast cancer tissues and cells. After its overexpression, MCF-7 cell apoptosis was significantly promoted. Furthermore, our data showed that miR-33-5p elevated apoptosis-related protein expression in MCF-7 cells. Contrary to hsa-miR-33-5p, SelT was upregulated both in breast cancer tissues and cells. SelT expression was significantly inhibited by hsa-miR-33-5p overexpression. The luciferase reporter assay confirmed that SelT was a direct target of hsa-miR-33-5p. SelT overexpression could ameliorate the increase in apoptosis induced by hsa-miR-33-5p mimics.

**Conclusion:** Our findings revealed that hsa-miR-33-5p, as a potential therapeutic target, could accelerate breast cancer cell apoptosis.

## Introduction

Breast cancer is a commonly diagnosed malignancy worldwide (1). Despite considerable progress in early detection and diagnosis, breast cancer patients' prognosis has been only slightly improved (2, 3). Moreover, its incidence in developed countries is still high, while it is increasing in developing countries due to changes in lifestyle and life expectancy (4). Hence, it is of significance to explore novel and effective therapeutic targets for breast cancer.

miRNA is a small and evolutionarily conserved non-coding RNA, with about 18–25 nucleotides in its length (5, 6). It has been widely accepted that miRNAs can bind to the 3′ untranslated region (UTR) of target genes, thereby negatively regulating their expression and causing the degradation of target mRNAs (7–9). It has been discovered that miRNAs are involved in many critical cell processes by regulating target genes at a post-transcriptional level, including cell apoptosis (10–12). There is ample evidence that aberrantly expressed miRNAs could be associated with breast cancer progression (13–15). So far, miRNAs have become promising markers for breast cancer due to the fact that they can be easily detected in tumor biopsies or body fluids (16). Dysregulated miRNAs have been recognized as early indicators and pathogenic factors of breast cancer (7, 17, 18). Furthermore, miRNA expression can predict the prognosis and progression of breast cancer (19–21). Therefore, a deeper understanding of miRNAs may provide opportunities for novel treatment strategies for breast cancer.

Previous studies found that hsa-miR-33-5p could induce osteoblast differentiation through targeting Hmga2 (22). Apoptosis is an important cellular process that is controlled by a variety of factors, including miRNAs. Abnormal apoptosis exhibits a close relationship with breast cancer occurrence, yet the function of hsa-miR-33-5p in breast cancer cell apoptosis needs to be clarified. In this study, our results suggested that hsa-miR-33-5p expression was downregulated in breast cancer. More importantly, its overexpression could promote breast cancer cell apoptosis. By further analysis, SelT could be directly targeted by hsa-miR-33-5p. Thus, our study proposed that hsa-miR-33-5p, as an underlying therapeutic target, could facilitate breast cancer cell apoptosis by SelT.

## Materials and Methods

### Tissue Specimens

Breast cancer tissues as well as corresponding normal tissue specimens were harvested from 20 patients with breast cancer from January 2018 to December 2019 in the Linyi Cancer Hospital. All tissues were instantly stored in liquid nitrogen. No patient received chemotherapy or radiation before surgery. All the patients provided written informed consent. The study was approved by the Ethics Committee of Linyi Cancer Hospital (LYZLYY-2018-012).

### Bioinformatics Analysis

The MiRWalk 2.0 database (http://mirwalk.uni-hd.de/) was used to predict the targets of hsa-miR-33-5p, which provides predicted and validated miRNA–target interactions (23). Furthermore, the immunohistochemical results of apoptosis-related proteins including Bax, Bcl2, caspase-3/8/9, and SelT in breast cancer and normal samples were downloaded from the Human Protein Atlas website (https://www.proteinatlas.org/).

### Cell Culture and Transfection

Human breast cancer MCF-7 and MDA-MB-231 cells and normal breast MCF10A cells were grown in 1640 medium (Invitrogen, CA, USA) with 10% FBS at 37°C and 5% CO_2_ in a humid environment. hsa-miR-33-5p mimics, siRNAs against SelT, and corresponding controls were used for transfection into two cells *via* Lipofectamine 2000 (Invitrogen). At 48 h after transfection, the cells were collected for further analysis. The cultured cells were treated with H_2_O_2_ as a control.

### Flow Cytometry

Cellular apoptotic levels were examined *via* the annexin V-FITC/PI apoptosis detection kit (Keygene, Nanjing, China). At 48 h after transfection, the cell resuspension (100 μl) was treated with annexin V-FITC/PI lasting for 15 min in the dark. The apoptotic rates were detected by flow cytometry.

### RT-qPCR

Total RNA was extracted using TRIzol (Invitrogen), followed by reverse transcription into cDNAs. The RT-qPCR was carried out on the real-time PCR system. The primer sequences of hsa-miR-33-5p and SelT were as follows: hsa-miR-33-5p, 5′-GGAMCTWYACGVAGGTG-3′ (forward), 5′-TGAAMTGCACRGAGCTTGC-3′ (reverse); SelT, 5′-TTGCTGCTTCTGCTGGTG-3′ (forward), 5′-CGTGGCGTACTGCATCTT-3′(reverse); β-actin, 5′-CGAGAAGATGACCCAGATCATG-3′ (forward), 5′-GTGAAGCTGTAGCCGCGCTCGG-3′ (reverse). β-actin was used as a control. Then, their expression levels were determined with the 2^−ΔΔCT^ method.

### Western Blot

Protein was extracted *via* RIPA lysis buffers (Beyotime, China), which was assessed with a bicinchoninic acid protein assay kit (Beyotime). The sample was separated through sodium dodecyl sulfate polyacrylamide gel electrophoresis and transferred onto a PVDF membrane. Then, the membrane was blocked using 5% non-fat milk for 1 h, which was incubated overnight with primary antibodies at 4°C and secondary antibody (1:2,000; Abcam, USA) lasting 1 h at room temperature. The primary antibodies included anti-Bax (1:1,000; Abcam), anti-Bcl2 (1:1,000; Abcam), anti-cleaved-caspase-3 (1:1,000; Abcam), anti-pro-caspase-3 (1:1,000; Abcam), anti-cleaved-caspase-8 (1:1,000; Abcam), anti-pro-caspase-8 (1:1,000; Abcam), anti-cleaved-caspase-9 (1:1,000; Abcam), anti-pro-caspase-9 (1:1,000; Abcam), and anti-GAPDH (1:1,000; Abcam). GAPDH served as a control. Image Lab™ Software (Bio-Rad, China) was used to quantify the intensity of blots.

### Luciferase Reporter Assay

The wild-type luciferase vector (wt-LucSelT) containing hsa-miR-33-5p response elements in the 3′UTR of SelT, or the mutant (mut-LucSelT) vector was constructed and transfected in RKO cells with hsa-miR-33-5p mimics or its control. Luciferase reporter assay systems (Promega, USA) were utilized to quantify the firefly and Renilla luciferase activity.

### Transferase-Mediated dUTP Nick End Labeling Staining

A transferase-mediated dUTP nick end labeling (TUNEL) kit (Atagenix, Wuhan, China) was used to assess the apoptotic levels. The sections were treated with TUNEL solution in the dark for 60 min. Afterwards, the samples were incubated with 0.05 μg/μl of 4′,6-diamidino-2-phenylindole solution for 10 min. Anti-fluorescence quenching mounting tablets were utilized for mounting. Images were investigated under a fluorescence microscope (Olympus, Japan).

### Statistical Analyses

Statistical analyses were performed using GraphPad Prism 8.0. Data are expressed as mean ± standard deviation from at least three independent experiments. Comparison between different groups was analyzed using Student's *t* test or one-way analysis of variance. *P* < 0.05 was considered statistically significant.

## Results

### hsa-miR-33-5p Is Downregulated in Breast Cancer and Its Overexpression Promotes the Apoptosis of Breast Cancer Cells

**Figure 1:**
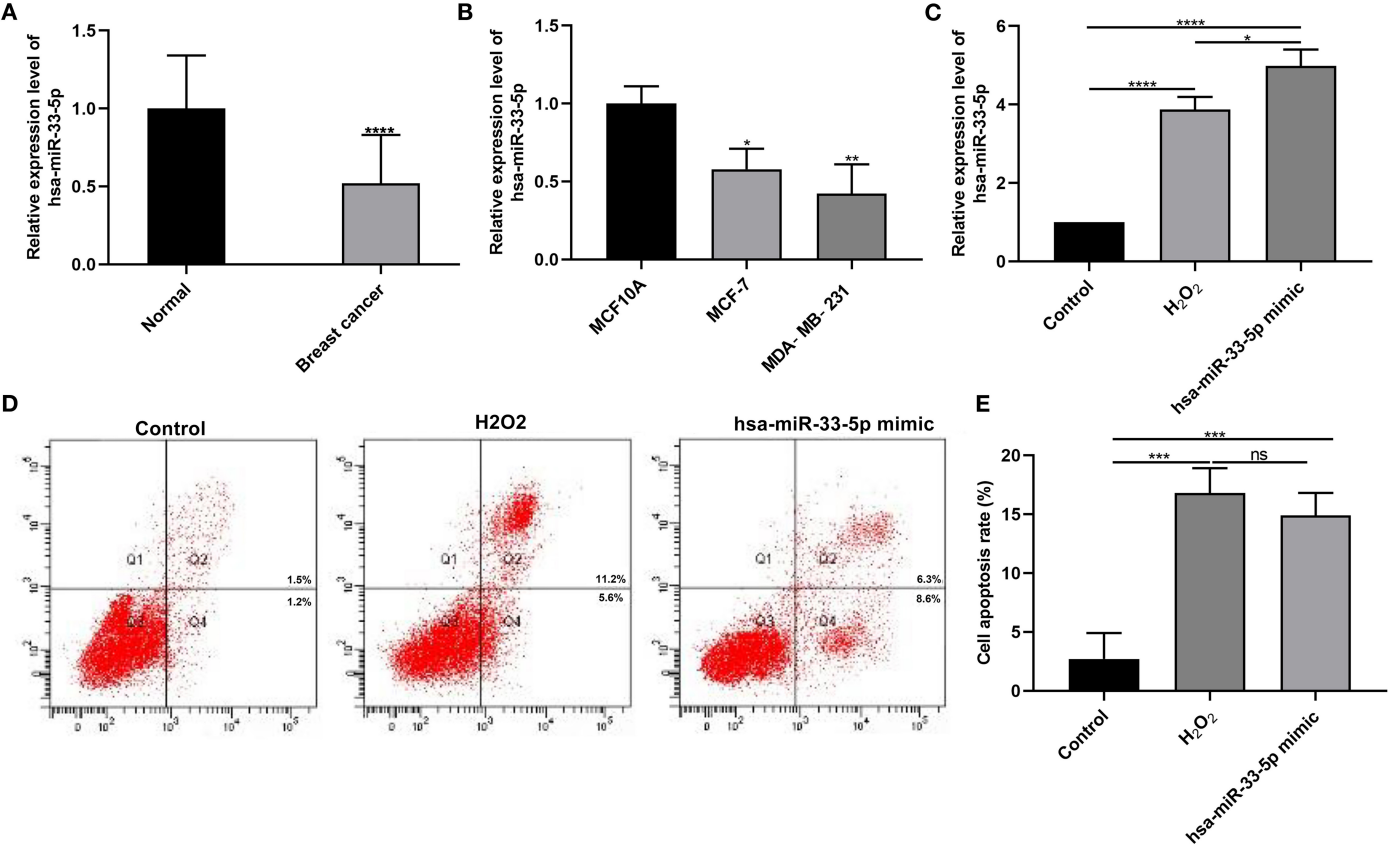
hsa-miR-33-5p is downregulated in breast cancer, and its overexpression promotes the apoptosis of breast cancer cells. **(A,B)** RT-qPCR results showing downregulated hsa-miR-33-5p both in breast cancer tissues **(A)** and cells **(B)**. **(C)** RT-qPCR showing hsa-miR-33-5p expression in MCF-7 cells following transfection by hsa-miR-33-5p mimics. **(D,E)** Flow cytometry for the cell apoptosis in MCF-7 cells under transfection by hsa-miR-33-5p mimics. *p < 0.05; **p < 0.01; ***p < 0.001; ****p < 0.0001; ns, not significant.

According to RT-qPCR results, hsa-miR-33-5p expression was lower in breast cancer than normal tissue specimens (Figure 1A; *p* < 0.0001). Furthermore, its lower expression was observed in MCF-7 as well as MDA-MB-231 cells more than MCF10A cells (both *p* < 0.05; Figure 1B). These results suggested that hsa-miR-33-5p was downregulated in breast cancer tissues as well as cells. As shown in Figure 1C, hsa-miR-33-5p mimics significantly overexpressed its expression compared to control (*p* < 0.0001). We also found that H_2_O_2_ significantly elevated hsa-miR-33-5p expression in MCF-7 cells (Figure 1C; *p* < 0.0001). hsa-miR-33-5p expression was significantly higher in MCF-7 cells following transfection by hsa-miR-33-5p overexpression than H_2_O_2_ treatment (Figure 1C; *p* < 0.05). The above-mentioned results suggested that hsa-miR-33-5p was successfully overexpressed. The flow cytometry results suggested that hsa-miR-33-5p overexpression could promote the apoptosis of MCF-7 cells (Figures 1D,E; *p* < 0.001). Furthermore, compared to control, H_2_O_2_ treatment also significantly induced cell apoptosis (*p* < 0.001). However, there was no significant difference in cell apoptosis between cells transfected with hsa-miR-33-5p mimics and H_2_O_2_ treatment.

### hsa-miR-33-5p Significantly Mediates Apoptosis-Related Proteins in Breast Cancer Cells

**Figure 2:**
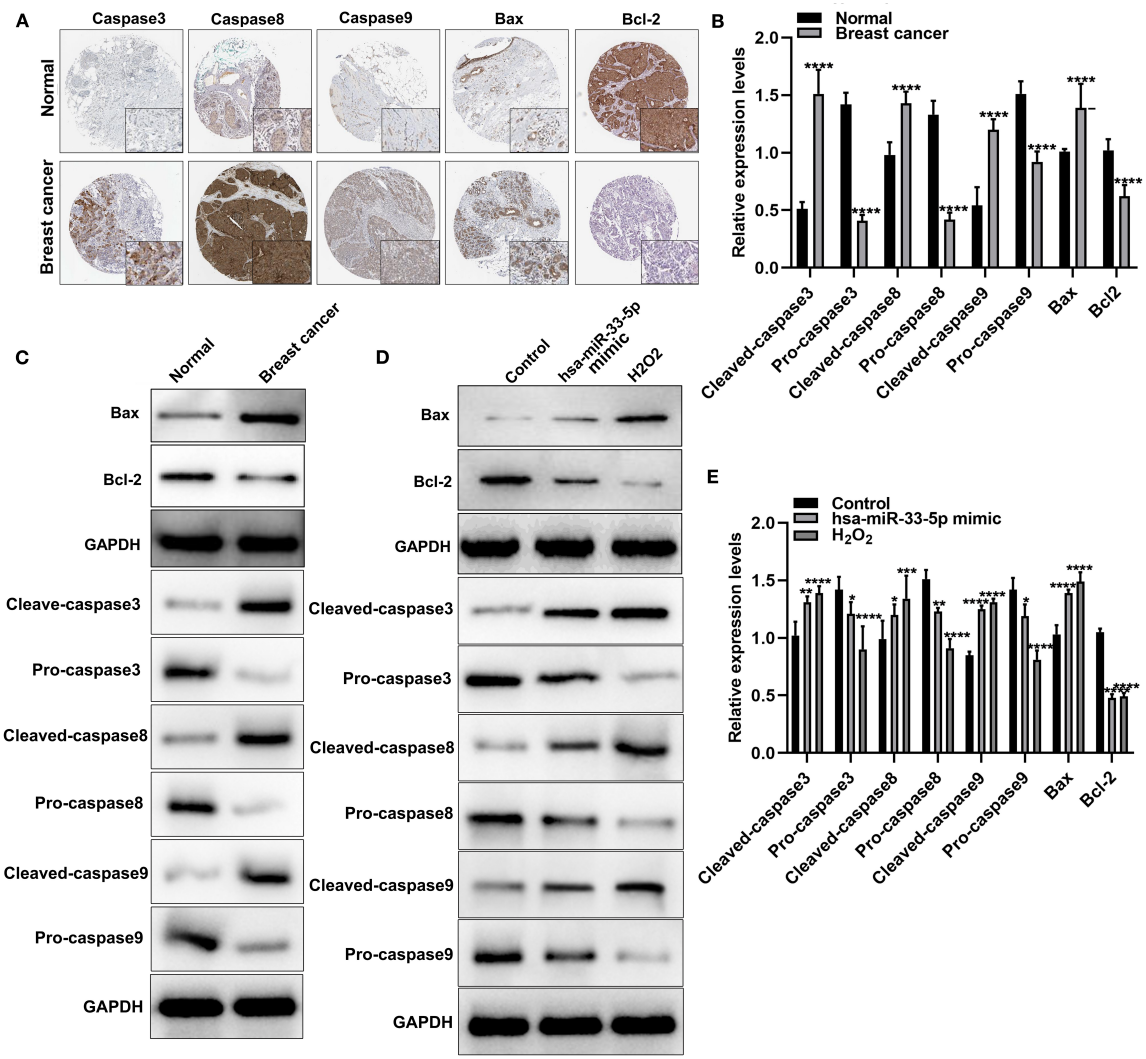
hsa-miR-33-5p significantly mediates apoptosis-related proteins in breast carcinoma cells. **(A)** Immunohistochemistry for the expression and distribution of the apoptosis-related proteins including caspase-3/8/9, Bax, and Bcl-2 in breast cancer tissues and normal tissues. **(B,C)** Western blot for the expression of apoptosis-related proteins including cleaved caspase-3, pro-caspase-3, cleaved caspase-8, pro-caspase-8, cleaved caspase-9, pro-caspase-9, Bax, and Bcl-2 in breast cancer tissues and normal tissues. **(D,E)** Western blot for the expression of cleaved caspase-3, pro-caspase-3, cleaved caspase-8, pro-caspase-8, cleaved caspase-9, pro-caspase-9, Bax, and Bcl-2 in breast cancer cells transfected with hsa-miR-33-5p mimics. *p < 0.05; **p < 0.01; ***p < 0.001; ****p < 0.0001.

Apoptosis is a key cellular process in breast cancer. We detected apoptosis-related proteins in tumor tissues. Figure 2A depicts the expression and distribution of these apoptosis-related proteins including caspase-3, caspase-8, caspase-9, Bax, and Bcl-2 in breast cancer tissues according to immunohistochemistry results. Furthermore, using western blot, we found that cleaved caspase-3, cleaved caspase-8, cleaved caspase-9, and Bax proteins were significantly overexpressed in breast cancer and normal tissues (all *p* < 0.0001; Figures 2B,C). Meanwhile, pro-caspase-3, pro-caspase-8, pro-caspase-9, and Bcl-2 protein (all *p* < 0.0001) exhibited significantly low expressions in breast cancer tissues (Figures 2B,C). hsa-miR-33-5p overexpression significantly elevated the expression of cleaved caspase-3 (*p* < 0.01), cleaved caspase-8 (*p* < 0.05), cleaved caspase-9 (*p* < 0.0001), and Bax (*p* < 0.0001) compared to control (Figures 2D,E). Moreover, pro-caspase-3 (*p* < 0.05), pro-caspase-8 (*p* < 0.01), pro-caspase-9 (*p* < 0.05), and Bcl-2 (*p* < 0.0001) expressions were distinctly lowered in MCF-7 cells after transfection by overexpressed hsa-miR-33-5p (Figures 2D,E). Similar results were investigated in MCF-7 cells exposed to H_2_O_2_. Thus, hsa-miR-33-5p could mediate apoptosis-related proteins in breast cancer cells.

### SelT Is Upregulated in Breast Carcinoma and Is a Target of hsa-miR-33-5p

**Figure 3:**
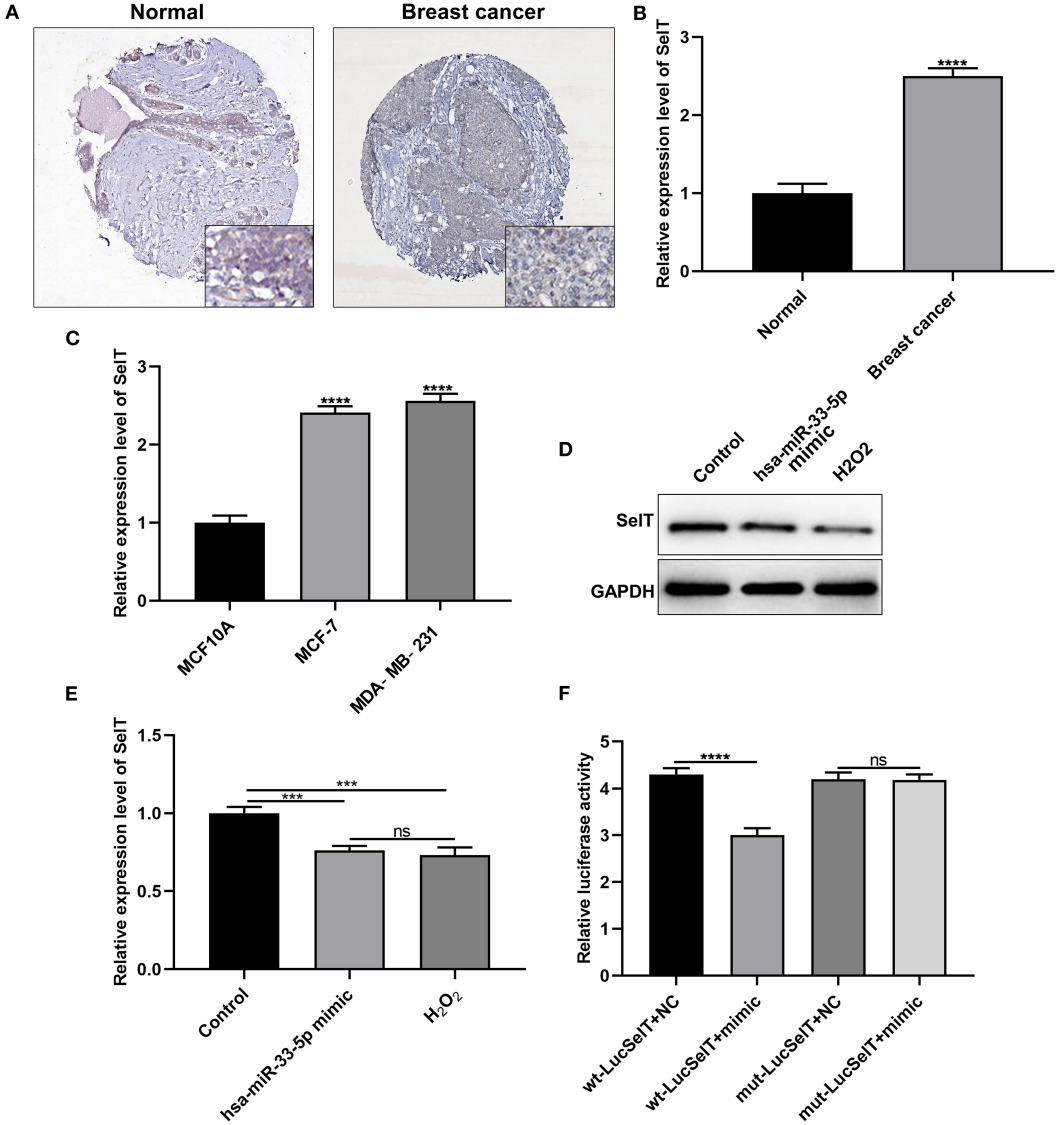
SelT is upregulated in breast carcinoma and is an underlying target of hsa-miR-33-5p. **(A)** Immunohistochemistry for the expression of SelT in breast cancer and normal tissues. **(B,C)** RT-qPCR results showing upregulated SelT both in breast cancer tissues **(B)** and cells **(C)**. **(D,E)** Western blot results for the expression of SelT in breast cancer cells transfected with hsa-miR-33-5p mimics or treated with H_2_O_2_. **(F)** Luciferase reporter assay for hsa-miR-33-5p and SelT. ***p < 0.001; ****p < 0.0001; ns, not significant.

As demonstrated by immunohistochemistry and RT-qPCR results, SelT expression had a significantly higher level in breast carcinoma than normal tissue samples (Figures 3A,B; *p* < 0.0001). Similarly, its upregulation was found in MCF-7 and MDA-MB-231 cells more than MCF10A cells (Figure 3C; *p* < 0.001). The above-mentioned results confirmed the upregulation of SelT in both breast cancer tissues and cells. As predicted, SelT could be a target of hsa-miR-33-5p. As shown in Figures 3D,E, SelT was significantly suppressed in breast cancer cells following transfection by overexpressed hsa-miR-33-5p or H_2_O_2_ treatment at the protein level (*p* < 0.001). However, no statistical difference in SelT expression was observed between hsa-miR-33-5p overexpression and H_2_O_2_ treatment groups. The luciferase reporter assay results confirmed that SelT was directly targeted by hsa-miR-33-5p (Figure 3F).

### Inhibition of SelT Promotes the Apoptosis of Breast Cancer Cells

**Figure 4:**
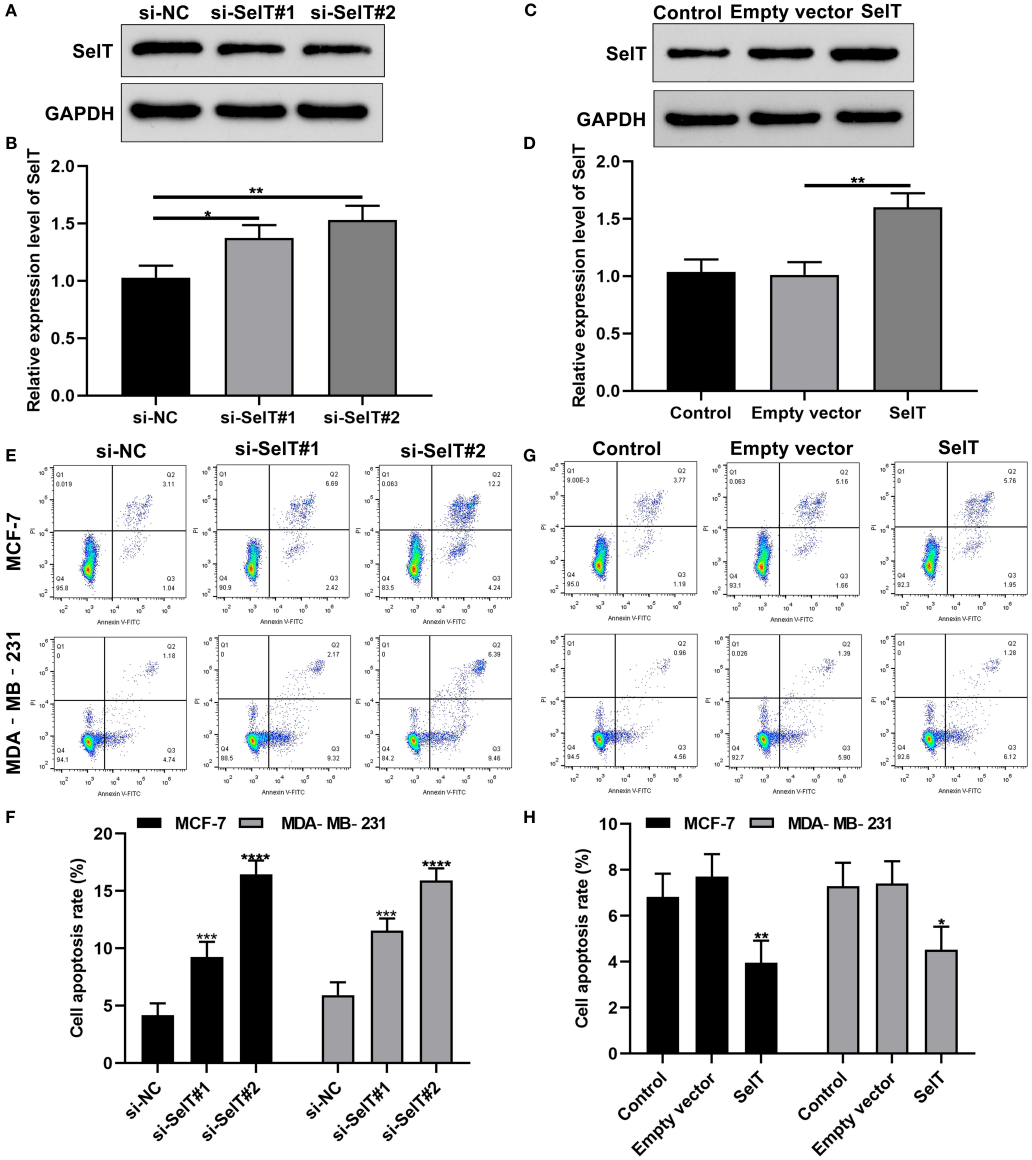
Inhibition of SelT promotes the apoptosis of breast cancer cells. **(A,B)** Western blot for the expression of SelT in breast cancer cells transfected with si-SelT#1 and si-SelT#2. **(C,D)** Validation of SelT expression in breast cancer cells transfected with SelT by western blot. **(E,F)** Flow cytometry assay for apoptotic levels of MCF-7 as well as MDA-MB-231 cells transfected by si-SelT#1 and si-SelT#2. **(G,H)** Assessment of breast carcinoma cell apoptosis under SelT overexpression. *p < 0.05; **p < 0.01; ***p < 0.001; ****p < 0.0001.

We further investigated whether SelT could promote the apoptosis of breast cancer cells. Two siRNAs targeting SelT were designed and transfected into breast cancer cells. As shown in Figures 4A,B, SelT expression was significantly suppressed by si-SelT#1 (*p* < 0.05) and si-SelT#2 (*p* < 0.01). Furthermore, SelT was successfully overexpressed in breast cancer cells (Figures 4C,D; *p* < 0.01). The flow cytometry data demonstrated that SelT knockdown markedly elevated the apoptotic levels of MCF-7 as well as MDA-MB-231 cells (Figures 4E,F). Meanwhile, SelT overexpression distinctly inhibited the apoptosis of MCF-7 as well as MDA-MB-231 cells (Figures 4G,H). The data demonstrated that inhibition of SelT may promote the apoptosis of breast cancer cells.

### hsa-miR-33-5p Promotes the Apoptosis of Breast Cancer Cells *via* SelT

**Figure 5:**
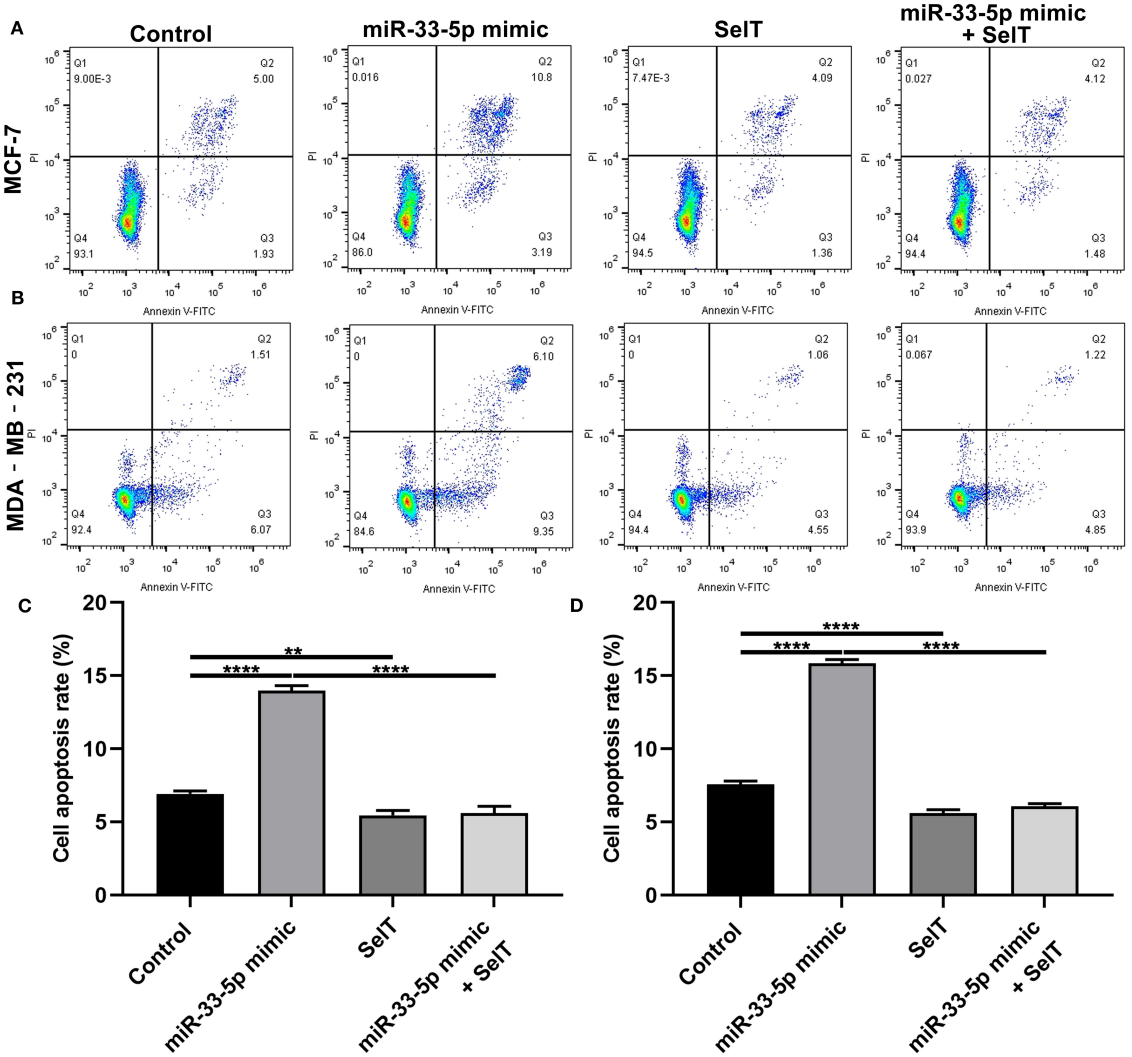
hsa-miR-33-5p promotes the apoptosis of breast cancer cells *via* SelT. **(A,B)** Representative images of flow cytometry assay results. **(C)** MCF-7 as well as **(D)** MDA-MB-231 cell apoptosis was determined when transfected by hsa-miR-33-5p mimics and/or SelT. **p < 0.01; ****p < 0.0001.

**Figure 6:**
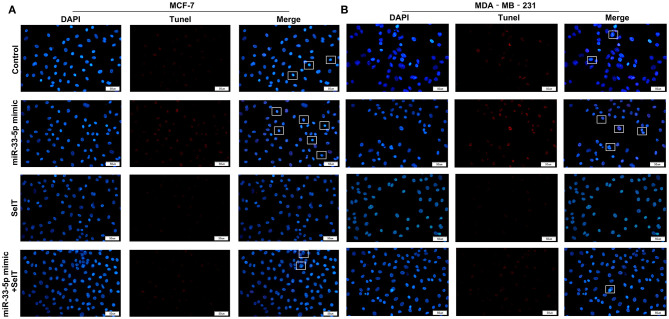
TUNEL staining for **(A)** MCF-7 as well as **(B)** MDA-MB-231 cell apoptosis transfected by hsa-miR-33-5p mimics and/or SelT. Bar = 50 μm. Magnification, ×400.

Our further analysis found that hsa-miR-33-5p mimics promoted the apoptosis of MCF-7 (*p* < 0.0001) and MDA-MB-231 cells (*p* < 0.0001), which was significantly ameliorated by SelT overexpression (*p* < 0.0001), as shown in Figures 5A–D. Furthermore, TUNEL staining results confirmed that SelT overexpression distinctly ameliorated the increase in apoptosis induced by hsa-miR-33-5p mimics in MCF-7 (Figure 6A) and MDA-MB-231 (Figure 6B) cells. These data demonstrated that hsa-miR-33-5p may promote the apoptosis of breast cancer cells *via* SelT.

## Discussion

In these findings, we identified a novel miRNA, and found that hsa-miR-33-5p was downregulated in both breast cancer tissues and cells. Its overexpression significantly promoted the apoptosis of breast cancer cells. Furthermore, SelT was an underlying target of hsa-miR-33-5p in breast cancer. hsa-miR-33-5p could facilitate the apoptosis of breast cancer cells *via* SelT. Our findings deepened the understanding of molecular mechanisms in breast cancer progression and provided potential therapeutic targets.

Our findings demonstrated that hsa-miR-33-5p was dysregulated in breast cancer. So far, no studies have reported the expression and role of hsa-miR-33-5p in breast cancer. As previously reported, hsa-miR-33-5p could induce osteoblast differentiation through targeting Hmga2 (22, 24). Its knockdown could inhibit abdominal aortic aneurysm development through ABCA1 expression and activation of the PI3K/Akt pathway (25). Lowly expressed hsa-miR-33-5p is involved in inhibiting the apoptosis of murine dorsal root ganglion neurons (26). Furthermore, hsa-miR-33-5p is in association with mesangial cell apoptosis in diabetic nephropathy (27).

Resistance to apoptosis is one of the hallmarks of cancer (28). Apoptosis could maintain homeostasis *via* mediating senescent cell death (29). Tumor cells can resist apoptosis by upregulating anti-apoptotic proteins and/or reducing pro-apoptotic proteins (30). Apoptotic caspases mainly include initiator caspases (caspase-2/8/9/10) and executioner caspases (caspase-3/6/7) (31). Bax is an apoptotic protein, and Bcl2 is an important regulator of anti-apoptosis, both of which are involved in mitochondrial death signals (32). Thus, inducing apoptosis of tumor cells has a promising potential to eradicate cancer cells. In this study, we examined apoptosis-related proteins including caspase-3/8/9, Bax, and Bcl-2. We found that caspase-3/8/9 and Bax expressions were elevated in breast cancer tissues, while Bcl-2 expression was decreased in breast cancer tissues. These findings revealed that the apoptosis process could occur in breast cancer. As with previous findings, many miRNAs could participate in breast cancer cell apoptosis. For example, hsa-miR-106a (33), hsa-miR-205 (34), and hsa-miR-216a (35) could induce the apoptosis of breast carcinoma cells. We explored the regulatory effect of hsa-miR-33-5p in breast cancer. hsa-miR-33-5p overexpression was successfully induced in MCF-7 cells. Its overexpression significantly induced the apoptosis of breast carcinoma cells as shown in flow cytometry results. Oxidative stress is involved in the process of apoptosis. H_2_O_2_ is one of the major components of exogenous reactive oxygen species, which is considered to be a key factor in regulating tumor cell viability (36). Different cell types have different responses to H_2_O_2_-induced oxidative stress and cell viability both in a dose- and time-dependent manner (37). As previously described, in this study, H_2_O_2_ exposure significantly induced breast cancer cell apoptosis. Furthermore, H_2_O_2_ exposure promoted hsa-miR-33-5p expression in MCF-7 cells. hsa-miR-33-5p overexpression significantly elevated caspase-3, caspase-8, caspase-9, and Bax expressions, while Bcl-2 was significantly inhibited in MCF-7 cells with hsa-miR-33-5p overexpression. As expected, similar results were investigated in MCF-7 cells exposed with H_2_O_2_. The above-mentioned data confirmed that hsa-miR-33-5p expression can be induced by oxidative stress.

Upregulated SelT was found in both breast cancer tissues and cells. Its knockdown could promote the apoptosis of breast cancer cells. SelT is a recently characterized thioredoxin-like protein that is widely expressed during development (38). It is upregulated during neuroendocrine cell differentiation (39, 40). Furthermore, SelT expression is strictly regulated in time. In most adult tissues, the expression level of SelT is decreased (41, 42). Previous studies have found that SelT is dysregulated in a few cancers like gastric cancer (43) and bladder cancer (44). hsa-miR-33-5p could significantly inhibit the expression of SelT. As validated by the luciferase reporter assay, SelT was directly targeted by hsa-miR-33-5p. hsa-miR-33-5p could accelerate the apoptosis of breast cancer cells *via* SelT. Furthermore, the results showed that, when breast cancer cells were exposed to H_2_O_2_, SelT expression was significantly inhibited. SelT could regulate various biological processes, like apoptosis. It possesses oxidoreductase functions and is localized in the endoplasmic reticulum. A similar result has found that, when LO2 cells were exposed to H_2_O_2_, SelX expression was suppressed and cell apoptosis was induced (45). The above-mentioned findings were indicative of the fact that SelT may participate in oxidative stress response and apoptosis in breast cancer, which was consistent with a previous study (46).

In conclusion, we found that hsa-miR-33-5p could induce the apoptosis of breast cancer cells. Moreover, SelT was directly targeted by hsa-miR-33-5p in breast cancer. hsa-miR-33-5p may facilitate the apoptosis of breast cancer cells *via* SelT. In future studies, we will continue studying the regulatory relationships between hsa-miR-33-5p and SelT and their functions on breast carcinoma cell apoptosis in animal models.

## Conclusion

In this study, downregulated hsa-miR-33-5p was investigated in breast cancer tissues and cells. Its overexpression could promote the apoptosis of breast cancer cells. Furthermore, SelT was targeted by hsa-miR-33-5p. These findings may offer novel therapeutic targets against breast carcinoma.

## Data Availability Statement

The datasets presented in this study can be found in online repositories. The names of the repository/repositories and accession number(s) can be found in the article/supplementary material.

## Ethics Statement

The studies involving human participants were reviewed and approved by The study was approved by the Ethics Committee of Linyi Cancer Hospital (LYZLYY-2018-012). The patients/participants provided their written informed consent to participate in this study.

## Author Contributions

WL conceived and designed the study. WZ conducted most of the experiments and data analysis and wrote the manuscript. JL participated in collecting data and helped to draft the manuscript. All the authors reviewed and approved the manuscript.

## Conflict of Interest

The authors declare that the research was conducted in the absence of any commercial or financial relationships that could be construed as a potential conflict of interest.

## Abbreviations

miRNAs: microRNAs
SelT: selenoprotein T
UTR: untranslated region.
